# Psychosocial workload and stress in the workers’ representative

**DOI:** 10.1186/1471-2458-12-909

**Published:** 2012-10-26

**Authors:** Martin Rabe, Salvatore Giacomuzzi, Matthias Nübling

**Affiliations:** 1 Institute for Public Health, UMIT, The Health and Life Sciences University Hall, Tyrol, Austria; 2 FFAS – Freiburg Research Centre of Occupational and Social Medicine, Freiburg, Germany

**Keywords:** Psychosocial workload, Industrial relations, Human resources, Trade union, Works councils

## Abstract

**Background:**

Using a data set of works councils of trade union IG Metal, this paper investigates psychosocial stress and strain on this specific group in comparison to employees working in administration in general (leadership and non-leadership-role) and a national reference value.

**Methods:**

For assessing psychosocial work factors on works councils within the sector represented by the trade union IG Metal in Germany, a research by using the German standard version of COPSOQ (Copenhagen Psychosocial Questionnaire) was performed. The instrument includes 87 single items forming 25 aspects of strain and stress. Results from the study group of works councils were compared to those from employees working in administration and to the general population mean (COPSOQ database). Statistical analysis included t-tests, analysis of variance and multiple comparisons of means. To be significant in terms of statistics, p<0.05 (two-tailed) and a minimum deviation of 5 or more points between groups′ mean values identify the relevant values.

**Results:**

All in all, 309 works councils from a national survey of the German chemical and metalworking industries took part in the study. 113 were full-time works council members (exempted from the duty to perform their regular work), 196 were voluntary members (acting as employee representatives on an honorary basis alongside their normal duties). Comparison between works councils and employees working in administration (leadership roles (N=1810) and non-leadership roles (N=2970)) and for employees in general (N=35.000) showed unfavourable values for works councils for most scales. Significantly higher values indicating higher strain and stress were found for the scales: emotional demands, work-privacy conflict, role conflicts, mobbing, cognitive stress symptoms and burnout. Unfavourable results were obtained for the aspects: quality of leadership, social support, sense of community and general health. Favourable findings were found on the scales: influence at work, quantity of social relations and the partly positive values for quantitative demands and commitment to the workplace.

**Conclusion:**

Compared to the reference groups, works council members perceive the psychosocial demands of working life as more exhausting for the majority of aspects. This allows several conclusions. One reason may be the extended tasks employee representatives face, an other may be that the education of most works council members does not seem appropriate to the high demands of their managerial and executive tasks.

## Background

In the last decades western industrial countries have been subject to diverse, often substantial variations of working conditions [1]. The comprehensive changes to the production and service sectors are leading to changing demands on the employees. Flexibility and knowledge are increasingly becoming key qualifications. These changes also placing different demands on workforce representatives, i.e. works councils [2]. As the link between employers and employees in industrial relations, works councils have to mediate between employer and employee interests. This role requires mental strength, competence and control, while helplessness and failure are perceived as disadvantageous [3,4]. Works councils mark a key institutional difference to other systems of workplace representation because they are formally independent of trade unions in their role as the worker′s representative [5]. They are endowed with a sophisticated system of codetermination rights and they negotiate company-based agreements without interfering with the collective bargaining agreements negotiated by the trade unions [6]. Taken together, these constitute the cornerstone of the dual system of industrial relations in Germany and differ from the Anglo-Saxon company-level bargaining or the Scandinavian countries′ centralised system of collective bargaining with union representations at the workplace [6], p.1.

This study, however, deals with the measurement of psychosocial stress and strain.

In the last decades a lot of scientific researches investigated and confirmed the relationship between job characteristics and employee′s health. They can have a substantial influence on physical and mental well-being, for example burnout, job strain or coronary heart disease. Thus, Doi [7] shows that job strains like high pressure of work, role ambiguity and emotional demands can lead to exhaustion and sleeping problems. On the other hand, motivational processes may arise through job resources, for example assistance or feedback on own performance [8,9] or training courses. Even if the dual system of industrial relations in Germany differs from many European countries, the research about the relationship of the “voice” of employees and increasing productivity and well-being at work indicating equivalent results, so the “Workplace Employment Relation Survey” (WERS) in the UK, the “RElations PrOfessionelles et NégociationS d′Entreprise” (REPONSE) in France and the “Work Councils Survey” (WSI Betriebsrätebefragung) in Germany [10-12]. Taking into account that the central role of work councils in industrial relations is well analysed, the authors found that, to the best of their knowledge, no recent research of cross-sectional studies which analyse psychosocial stress and strain on this profession. There is research, e.g. Korunka et al. [13] comparing white and blue-collar workers and different groups of employees, but not employee representatives.

In a first step, the authors analysed different questionnaires to identify an instrument which was firstly sensitive enough to measure stress and strain, secondly addressed the demands and other workplace factors faced by this specific group and thirdly allowed a comparison of works councils both to employees working in similar jobs and employees in general. The authors decided to measure the psychosocial work environment by the use of Copenhagen Psychosocial Questionnaire (COPSOQ) [14].

COPSOQ is a relatively new established, broad and comprehensive questionnaire. According to the author Kristensen, it is “theory-based, but not attached to one specific theory”. He further states that for reasons of content validity such a tool “ …should include dimensions related to work tasks, the organization of work, interpersonal relations at work, cooperation and leadership, …[and] should cover potential work stressors, as well as resources” [15], p. 439. This comprehensive instrument “not only measures specifically defined potentially health-hazardous constellations at work […], but has the objective of assessing all relevant aspects of the psychosocial work environment” [16], p.3.

### Theory

Generally, the measurement of psychological stress refers to recent stress models.

In work science there are different factors indicating psychological stress. There are measurable external factors like workload und time pressure, internal factors depending on an individual’s own conditions, and the consequences, such as disease. The common factor shared by these is “that job strain is the result of a disturbance of the equilibrium between the demands employees are exposed to and the resources they have at their disposal” [17], p. 310.

Over recent decades key models such as the demand-control model (DCM), the effort-reward-imbalance model (ERI) and the job demands-resources model (JD-R) have become essential for work science.

The DCM was devised by Karasek [18]. To get a substantial description of reality, the model was extended by the dimension of social support to the demand-control-support model [19]. The model describes stress and strain of working life as a consequence of the imbalance between individual resources and the professional requirements. So the situation of high demands at work, for example time pressure or overwork and a low degree of influence on job is responsible for the emergence of stress.

It is a cornerstone of the DCM that independent decision latitude of employees has a preventive effect on stress and strain at work. Karasek states [20], p. 287:

“The individual’s decision latitude is the constraint which modulates the release or transformation of “stress” (potential energy) into the energy of action.”

Scientific literature supports the hypotheses of DCM, but it has to be said that De Jonge and Kompier [21] confirm less consistency.

Driven from another perspective, the effort-reward imbalance (ERI) model was formulated by Siegrist [22,23]. It focuses the meaning of rewards on performed work and health. So high performance on job combined with fewer prospects of rewards lead to adverse effects, for example less well-being. In principle according to this theory an imbalance between efforts compared to rewards leads to excitation and stress, which may cause cardiovascular risk and further strain reactions [21,24]. This hypothesis is fostered by Van Vegchel et al. in a review of 45 studies [25]. In contrast to the DCM, the ERI model takes into account that personal components are important as well. As the model indicates, engagement and commitment could mediate the relationship between the disequilibrium of efforts and rewards and employees′ general health [26].

Both the ERI model and the DCM assume that profession demands lead cause stress and strain reactions [27]. It should be mentioned that a main point of criticism of the models is their static character. Additionally, it is contestable whether autonomy is the most precious resource for employees [28] in the DCM, whereas it is a point of discussion that the ERI model fosters salary and esteem rewards as the main means of compensating job strain. So the advantage of the models, their simplicity, is also their weakness because they do not cover the complex reality. Karasek [20], p. 290 himself acknowledged a wider range of job demands and a more centred role of resources:

*In future research it would be desirable to discriminate between the effects of several different aspects of decision latitude* (*i*.*e*. *with respect to skill*, *task organization*, *time pacing*, *organizational policy influence*, *control over potential uncertainties*, *decision resources*). 

**Figure 1:**
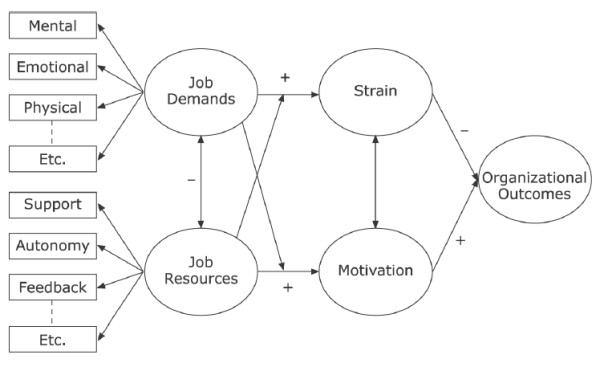
**The Job Demands-Resources Model **[20]**, p.313.**

Combining both models, the job demands-resources (JD-R) model by Baker implies that “every occupation may have its own specific risk factors associated with job stress, these factors can be classified in two general categories (i.e. job demands and job resources), thus constituting an overarching model that may be applied to various occupational settings, irrespective of the particular demands and resources involved” [17], p. 312. Job demands describes the parts of working day where persistent mental or physical efforts arise. These requirements lead to corresponding mental or physical costs. Job resources describe processes which are supportive to deal with everyday work. The model describes psychosocial stress and strain as an outcome of high demands in working life and minor resources and reciprocally to less psychosocial stress and strain. Meijman et al. [29] state an interesting point of view. Even if the requirements of everyday working situation could imply a positive meaning, these demands could change into stress or strain if the employee is not adequately recovered to face them (Figure 1).

The COPSOQ questionnaire combines these theories and reveals advantages in use being “theory-based but not attached to one theory“ [15], p. 439. This comprehensive questionnaire was generated in Denmark by Kristensen and Borg [15,30] and adapted, tested and established in Germany by Nübling et al. [31,32]. In additional to the COPSOQ, the authors included questions on recent central and decentralised training courses by the trade union IG Metal in the questionnaire.

Based on the considerations above, the authors hypothesised that:

(1) Full-time and volunteer works councils perceive psychosocial stress and strain different

(2) Compared to other professions with partly matching tasks, work councils indicate disadvantageous values.

The aims of this research are to compare the psychosocial demands of works councils with selected groups. For comparison employees working in similar jobs (administration) and the average for all occupations in the COPSOQ database (profession-specific reference values from COPSOQ studies in Germany) were selected. Additionally, the second aim is analysing a general trend of percepting psychosocial stress and strain of these professions.

## Methods

### Instrument: the COPSOQ questionnaire coupled with questions on recent training courses by the trade union IG metal

For assessment of the psychosocial work factors on works councils, the authors used the German version of COPSOQ (Copenhagen Psychosocial Questionnaire). Kristensen and Borg in Denmark originally developed this comprehensive instrument in a Danish and English version for assessing psychosocial workload. The advantage of COPSOQ is that this questionnaire is “…theory-based but not attached to one specific theory” [15], p. 38. The German standard version was validated in 2003–2005 by Nübling et al. [31,32] and terms of the classification of methods measuring and assessing mental workload (ISO 10075–3) it is placed as a level 2 questionnaire. Level 1 questionnaires are used for precise measurement, level 3 are intended to provide guidance. Detailed information are available on the German COPSOQ website: http://www.copsoq.de.

**Figure 2:**
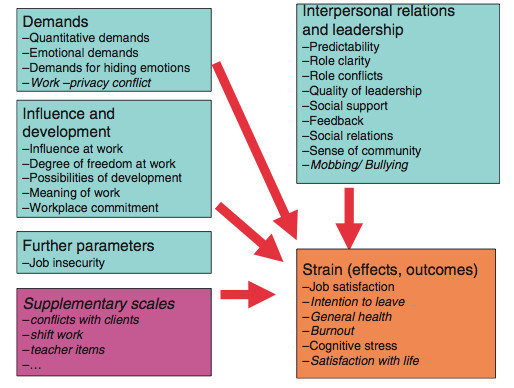
**Content of the German standard COPSOQ, differences from the Danish/English original questionnaire in italics **[33]**, p. 121.**

The COPSOQ uses 19 aspects to measure psychosocial demands at job environment [33], p.430. These aspects are divided into different segments. Four scales are used to measure “demands”, five scales to asses “influence and development”. Further there are eight scales and one single item measuring “interpersonal support and relationship” and one scale to asses “job insecurity”. Measuring the relationship and reaction of employees to their daily work, six subparts report as outcome factors on “job satisfaction, intention to leave, general health, burnout (scale: personal burnout), cognitive stress and satisfaction with life” [33], p.430. All in all the German standard version of COPSOQ contains 87 items. Most of them are based on a five-point Lickert scale. The structure and the suspected relation between job environment and the individuals′ implications are characterized by Figure 2.

The classification of occupations was grouped according to the assignment of job classifications KdB92 ("Klassifikation der Berufe 1992”) of the German Federal Statistical Office http://www.destatis.de.

It was not necessary to add specific questions for research on the profession of works (e.g. bargaining for working conditions). In principle the aspects of COPSOQ show values starting from the minimum 0, which means the least possible value and the maximum of 100, standing for the maximize indication. In order to present transparent and comprehensible results, the scale values are reported as mean values. The renunciation of presenting the values in percentages was done on purpose to avoid loss of information when combining sectors for calculating the percentage values.

The meaning of a outcome due to the indicated low or high values is related to the context. High values for “commitment to the workplace” or “meaning of work” indicate a positive meaning whereas high values for “quantitative demands” or “work-privacy conflict” indicate a disadvantageous meaning.

It is a great advantage of COPSOQ considering recent theories to measure psychosocial stress and strain at workplace as extensively as possible. According to Nübling et al. [32], p.3:

“*Therefore, the COPSOQ is covering a broad range of aspects of currently leading concepts and theories. The following are mentioned*[15]: “*1. the job characteristics model. 2. the Michigan organizational stress model. 3. the demand control-(support) model. 4. the sociotechnical approach. 5. the action theoretical approach. 6. the effort reward imbalance model. 7. the vitamin model.” The COPSOQ tries to deal with the broadness respectively indefiniteness of the construct “psychosocial factors” by applying a multidimensional approach with a very wide spectrum of ascertained aspects*.“

Additionally, the authors included questions on recent main training courses by IG Metall. These questions related to 10 main training courses including 48 specific seminars grouped into the sections: introduction (2 seminars), special training courses for works councils (6 seminars), special training courses for shop stewards (“Vertrauensleute”) (2 seminars), training for both works councils and shop stewards (2 seminars), task-released training (19 seminars), political education forum (3 seminars), social-political development (6 seminars), international education (2 seminars), political education for young people (4 seminars), economics ( 2 seminars). The authors requested that unlisted training courses be noted in free comments. It should be mentioned that questions related only to attendance, not to the quality of the courses.

### Performing the study on works councils

The cross-sectional survey was executed by the authors and supported by the trade union IG Metal. The board of directors of IG Metal approved the questionnaire in October 2011. In order to perform the survey, 50 administrative centres of the union send the questionnaire with a covering letter of the first author and a letter of recommendation from IG Metal to work councils committees via email and in a paper and pencil version. At the end of the survey period a reminder was send out. Participation was voluntary and anonymous; no personal data, such as company or address, were collected. Austrian and German ethic committee checked the need of ethical approval. The Research Committee for Scientific and Ethical Questions of UMIT (RCSEQ 143/12) and the Ethic Committee of Bavarian Medical Association (2012–032) stated that no ethical approval in line with the Declaration of Helsinki was required. Works councils sent their completed questionnaires in an anonymous envelope directly to the first author, some used a scanned version and sent it to the author by e-mail. Data were analysed in cooperation with the FFAS (Freiburg Research Centre for Occupational and Social Medicine). Statistical analysis was performed by SPSS 19.0 and involved descriptive statistics, t-tests and analysis of variance (ANOVA). The variety of tests (25 aspects and three comparisons to external reference values for each aspect) led to the establishment of significance at p < 0.05. In addition, a minimum distinction of means by five points or more set the lower limit for a significant difference. The standard deviations (SD) of the questionnaires’ scales are about 15–25 points, “a difference of 5 points corresponds to an effect size of at least 0.2 which is considered as being the threshold of a slight effect; for scales with smaller SDs the effect size is than 0.3-0.35 for a 5 point difference” [34], p.4.

## Results

Altogether, 309 works councils took part in the survey. The sizes of the companies employing participants varied greatly. So most of the participants are employed by companies with a workforce of 100 – 500 or over 2,000. Due to their occupation, participants were grouped: 113 are full-time works council members and 196 are volunteer works councils. The vast majority of participants in this survey are, as Table 1 shows, male. Furthermore, members aged 45 and older are well represented. Most of the participants work full-time, 20 of the 312 participants work between 15 and 34 hours per week and only 2 indicate a working time of 15 hours or less per week.

**Table 1:** Sociodemographic characteristics and working schedules of study participants

		**Total; N= 309**	
Gender	Male	225	72.80%
	Female	84	27.20%
Age	18-24 and 25 - 34	32	10.40%
	35-44	81	26.20%
	45-54	143	46.30%
	55+	47	15.20%
	No answer	6	1.19%
Working hours per week	35+ h/week	287	92.90%
	15-34 h/week	20	6.50%
	< 15 h/week	2	0.60%
Company size	0 - 100	33	10.70%
	100 - 500	90	20.10%
	500 - 1000	60	19.40%
	1000 - 1500	16	5.20%
	1500 - 2000	12	3.90%
	> 2000	98	31.70%
Kind of work	Mostly desk work	215	69.60%
	Mostly physically work	14	4.50%
	About equally	80	35.90%
	No answer	0	0.00%
Education	Secondary school certificate	4	1.30%
	Completed vocational training	237	76.70%
	College degree	21	8.70%
	No answer	41	13.30%
Kind of employee's representative	Full-time works council member	113	36.60%
	Volunteer works council member	196	56.00%
Industry	Metal-working	235	75.50%
	Service	36	11.70%
	IT	7	2.30%
	Chemical	18	5.90%
	Textile	8	2.60%
	Others	3	1.00%
KdB92 Classification	Storage management	11	3.60%
	Chemistry	4	1.30%
	Metal producing and working	29	9.40%
	Metal, machinist or similar	80	25.90%
	Electronics or similar	25	8.10%
	Machinist and engine driver	8	2.60%
	Engineer	20	6.50%
	Technical profession (engineer, chemist)	33	10.70%
	Service	21	6.80%
	Organisation and management (leadership role)	20	6.50%
	Business employees	47	15.20%
	Health sector	4	1.30%
	Social and educational profession	4	1.30%
	No answer	3	1.00%

Sum total may not be 100% due to rounding.

It has to be said as a weakness of this research that the authors were not able to measure the response rate of the questionnaire due to the fact that the administrative centres did not distributed information about the number or the structure of contacted work councils committees. The low number of participants could be explained by different reasons. One possible explanation for the relatively low number of responses is the effect, not predictable in advance, of the election of a new IG Metal board of directors at the end of October 2011. The free text statements on working conditions could also be an explanation: Many works councils indicated demotivation as a result of deterioration of working conditions in the companies (e.g. time pressure and increased work tasks). Another reason could be the questionnaire itself, its length means that it takes between 20 and 25 minutes to complete.

### Psychosocial factors at work

Internal comparisons for all 25 factors in the COPSOQ were performed between the group of works councils, employees working in similar jobs (administration) and also the mean value of all occupations of COPSOQ database (all professions, weighted mean according to the distribution of occupations in Germany N= 35,000 employees, available on http://www.copsoq.de, mostly in German). These data-sources were provided by FFAS and contain all results of research by COPSOQ in the period starting in Mai 2005 up to March 2012.

### Differences between works council members and external reference values from the COPSOQ database

Table 2 presents the results of the comparison between the scales of full time works councils (FWC), volunteer work councils (VWC), COPSOQ database reference value for administration and management (with a leadership role, LAD), administration and management (without a leadership role, NLA) and also the weighted mean value of the whole COPSOQ database (all occupations, ALL). Significant distinctions are at the level of p < 0.05 attained if the deviation of COPSOQ scales exceeds five points or more. In considering the mean values of works councils comparing to the selected groups the authors indicated variations of five points or more by using the “+”-sign. Vice versa, deviations of five points or less are indicated by using a “-“-sign. It should be noted that the indication of the signs does not imply a positive or negative meaning. The interpretation refers to the scales' content. In comparing the scale means between works councils, employees working in similar professions and the overall COPSOQ database value it is shown that works councils (indicated as “+”) have some significant disadvantages and advantages.

**Table 2:** Study results for psychosocial demands

**Scale and items**	**FWC, N = 113**		**VWC , N = 196**		**LAD , N = 1810**		**NLA , N = 2970**		**ALL , N > 35.000**		**FWC vs. LAD**	**VWC vs. LAD**	**FWC vs. NLA**	**VWC vs. NLA**	**FWC vs. ALL**	**VWC vs. ALL**
	**MW**	**(SD)**	**MW**	**(SD)**	**MW**	**(SD)**	**MW**	**SD**	**MW**	**SD**						
**Demands**																
Quantitative demands (high = pos.)	62	(15)	61	(16)	64	(19)	55	(19)	55	(19)			+	+	+	+
Emotional demands (high = pos.)	70	(16)	55	(19)	54	(20)	47	(22)	52	(22)	+		+	+	+	
Demands for hiding emotions (high = pos.)	47	(18)	45	(23)	49	(23)	45	(27)	46	(26)						
Work- privacy conflict (low = pos.)	53	(23)	47	(24)	44	(29)	34	(27)	42	(29)	+		+	+	+	+
**Influence and development**																
Influence at work (high = pos.)	57	(18)	45	(20)	41	(21)	34	(21)	42	(23)	+		+	+	+	
Degree of freedom at work (high = pos.)	71	(18)	60	(20)	63	(19)	58	(22)	53	(24)	+		+		+	+
Possibilities for development (high = pos.)	80	(13)	67	(20)	72	(17)	62	(19)	67	(20)	+	-	+	+	+	
Meaning of work (high = pos.)	85	(14)	67	(21)	71	(20)	70	(20)	74	(20)	+		+		+	-
Workplace commitment (high = pos.)	64	(14)	54	(19)	53	(20)	53	(20)	57	(20)	+		+		+	
**Interpersonal relations and leadership**																
Predictability (high = pos.)	62	(16)	48	(21)	53	(23)	50	(23)	54	(23)	+	-	+		+	-
Role clarity (high = pos.)	74	(13)	69	(16)	70	(19)	71	(18)	73	(18)						
Role conflicts low = pos.)	58	(18)	52	(19)	46	(21)	42	(21)	44	(21)	+	+	+	+	+	+
Quality of leadership (high = pos.)	22	(27)	43	(17)	49	(25)	49	(25)	50	(25)	-	+	-	-	-	-
Social support (high = pos.)	43	(20)	57	(16)	65	(20)	64	(23)	64	(22)	-	-	-	-	-	-
Feedback (high = pos.)	34	(17)	45	(16)	38	(21)	37	(22)	41	(22)		+		+	-	
Social relations (quantity) (high = pos.)	56	(19)	59	(19)	46	(27)	52	(27)	52	(28)	+	+		+		+
Sense of community (high = pos.)	69	(23)	71	(18)	77	(18)	75	(20)	75	(19)	-	-	-		-	
Mobbing (single item) (low = pos.)	29	(21)	40	(24)	18	(23)	20	(24)	21	(24)	+	+	+	+	+	+
**Additional scales**																
Job insecurity (low = pos.)	35	(17)	43	(16)	19	(17)	32	(22)	32	(24)	+	+		+		+
Intention to leave (single item) (low = pos.)	18	(21)	23	(28)	17	(24)	17	(24)	16	(23)		+		+		+
Job satisfaction (high = pos.)	66	(13)	56	(17)	64	(17)	62	(16)	63	(16)		-		-		-
General health (singel item) (high = pos.)	67	(20)	65	(21)	71	(19)	70	(20)	71	(20)		-		-		-
Personal burnout (low = pos.)	43	(17)	48	(20)	42	(20)	42	(20)	42	(19)		+		+		+
Cognitive stress symptoms (low = pos.)	35	(19)	40	(21)	29	(19)	30	(19)	29	(20)	+	+	+	+	+	+
Satisfaction with life scale (high = pos.)	68	(15)	65	(16)	67	(18)	65	(19)	65	(19)						

Differences in means of > = 5 points are indicated by “+": study group value work councils is higher than COPSOQ- database reference value for organisation and management (leading position) (LAD), organisation and management (no leading position) (NLA) or COPSOQ total (ALL) or by a “-": study group value > = 5 points worse than reference value. Remark: all differences reaching or exceeding +−5 points differences are significant with p < 0.05.

Scale means and standard deviation.

### Differences between works councils and external reference values from the COPSOQ database

Comparison of the study group with the general COPSOQ mean for all professions reveals both, significant and relevant deviations in some aspects. The significance value is at p <0.05, whereas relevance means **Δ** at minimum of 5 points or more. Indications for higher strain and stress values were found on the scales: emotional demands, work-privacy conflict, role conflicts, mobbing, cognitive stress symptoms and burnout. Unfavourable results were also obtained in the form of the lower mean values for works council members for the aspects: quality of leadership, social support, sense of community and general health.

From the perspective of works council members there are also positive findings in higher mean values for influence at work, quantity of social relations. There are relatively positive values for quantitative demands (compared to the general mean and to non-leadership administrative workers) and commitment to the workplace (compared to both groups of administrative workers).

### Trend of human being at work

**Figure 3:**
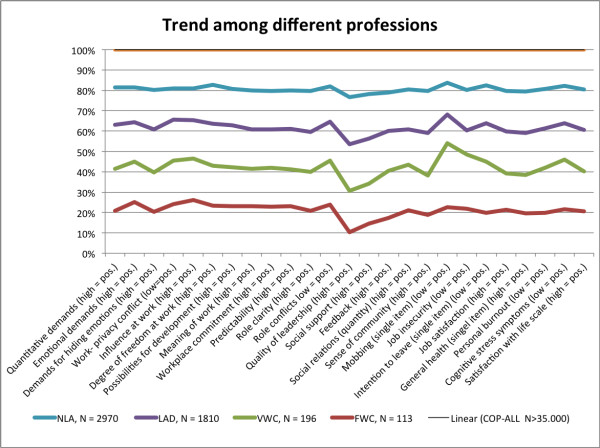
Relative trend among different professions, COPSOQ-ALL is set to 100%.

Analysing the different mean values it is interesting to reveal a general trend of psychosocial stress and strain among different professions and to take a closer view where there are similarities and differences. Figure 3 shows the trend of mean values compared to COPSOQ-ALL. It is to see that the profession of work councils proceed similar to employees working in the field of administration and management in a leading position but indicating higher deviations and in most of the scales higher demands for this profession.

## Discussion and conclusion

For measuring psychosocial work factors for works councils, the authors used the comprehensive and validated German version of COPSOQ. The great advantage of this questionnaire is the identification of different aspects of stress and strain and providing a comprehensive data setting enabling comparison of different professions. Due to the fact that work councils have complex tasks, the authors analysed the responsibilities of this specific profession. According to the assignment of job classifications KdB92 of the German Federal Statistical Office, the participation rights, intermediate functions and partly managerial tasks there are many comparable responsibilities to employees working in administration (leadership roles). Due to the fact that volunteer work councils represent the interests of employees in addition to their daily mostly mentally work, the authors decided to use the profession of employees working in administration in a non-leadership role as a further reference group.

The analysis of psychosocial workload of work councils and reference groups revealed new and interesting information. Due to the outcomes there is a general trend of psychosocial stress and strain among the analysed professions. This is not surprising, as the consequences of this trend is a part of the self-image of "Human being in profession".

A major finding is, by analysing the trend in detail it is to see that work councils proceed similarly to employees working in administration in a leadership role and confirm so the selection of reference professions. The authors confirmed presumed findings by comprehensive outcomes of psychosocial factors at work, for example the significantly higher scores in the study group for “emotional demands”, “work-privacy conflict” or “role conflicts” which are typical for this group as employee representatives and have been demonstrated in many other national and international surveys [3,5].

The second major finding of this research is that this study enables comparison between occupational groups and work councils and furthermore the determination of specific demands and deviations to reference groups. So there is a difference in psychosocial stress and strain within the profession of employees’ representatives. As one outcome, the perception of quality of leadership differs between volunteer and full-time work councils.

Volunteer work councils, being integrated in the workflow as “normal” employees, are organized in the subordination structure and perceive leadership of their disciplinary superiors positively. Full-time work councils form the opposite. They elect the chairman for a certain time and are subordinated on his decisions, but it is to say that there is no comparable setting to normal employees. It seems that the lack of framework of organisation leads to indication of disadvantageous values. Interesting is the outcome of the scales “mobbing” and “social support”. In both scales work councils are significant disadvantageous. Volunteer work councils state exceptional high strain on “mobbing” compared to full-time work councils but indicate to have a relatively positive social relations. This could be explained due to the integration of volunteer work councils into the working process. They are still “one of the employees”, while full-time work councils are not involved in the daily work, explaining the values of “social support”. The time volunteer work councils have to act as employees’ representatives, other workers have to take over their tasks and this leads to negative emotions, explaining the values of “mobbing”.

On a general level, the study also revealed a phenomenon we did not expect, namely the exceptionally high value for “job insecurity” because works councils are subject to the special protection of § 78 WCA (BetrVG), which means that they can only be dismissed by summary termination, § 15 EPA (KSchG). The summary termination must also be approved by the works council or – if the council does not agree - confirmed by the labour court. Due to the fact that the election period is four years, this limited period of protection may lead to a fear of reprisals and loss of employment after membership of the works council expires. Secondly, the work transition from representation back to “normal” work could be perceived as a disadvantage and might be an additional reason. Also workers who are afraid of losing their job might put themselves in a prime position for election.

The study results confirm the often reported high demands on works councils. The changing process of organised decentralisation poses new challenges for works councils in the system of industrial relations. Decentralisation leads to a loss of power for unions, because non-organised decentralisation leads to the use of derogations for a rising number of companies. This means a weakening of the protection through organised collective bargaining and shifts responsibilities from the level of collective bargaining to the level of plant bargaining, meaning to the profession of works councils. This leads to increasing emotional demands, high work-privacy conflicts and role conflicts in this complex, so called “triadic relationship” [3], p. 25.

The study results reveal positive findings on the scales: influence at work, degree of freedom, social relations, development and commitment. These findings were expected due to the fact that § 80 WCA empowers works council members. They can claim information and in managerial, technical and human resource decisions the management has to consult works councils. So works councils do have a significant influence on work, widespread social relations, are responsible for development and being elected to represent employee interests creates high commitment to the workplace by the necessary high degree of freedom (granted by WCA) to fulfil their tasks.

### Strengths and limitations

The major strength of this research is a comprehensive survey addressing the psychosocial workload within the profession of works councils. The extensive theoretical approach and design of this validated and psychometrically tested comprehensive questionnaire was used for the quantitative investigation of the psychosocial work factors in works councils. With more than 300 participants, this database is sufficient for an initial evaluation of their specific working situation. The results enable initial insights into the psychosocial workplace situation of this specific group to identify exact stress and strain factors. Furthermore, the outcome provides a comprehensive data setting for further research.

In line with Theorell et al. [27], the authors are conscious of limitations and shortcomings of researches performed by self-reported questionnaires. So the use of just a single data source is a general limitation. Furthermore, the collection of personal statements and reports on potential risk factors could lead to a “common method bias”. The cross-linking of different survey approaches and data settings on psychosocial workload would lead to advantages. Kompier calls this a “multi-source” assessment [35], which has been performed in some studies. So for example researches, matching “subjective” data sources by the use of COPSOQ and “objective” source by medical examination [33]. However, these multi-source studies require much greater resources and different assessment methods (such as a personal examination performed by a physician).

In the field of psychosocial factors, many aspects can only be assessed by subjective methods, asking the employees directly. We agree with Kompier that works councils are professionals on their job and are aware of the opportunities and threats for this occupation. The question of “objective” and “subjective” measurement is not the main dichotomy or quality criteria in measurement. The main point is whether the reliability, validity and quality of the questionnaire′s design, psychometric testing and adequate statistical analysis of the results are fulfilled.

It has to be said as a proviso that the authors were not able to measure a response rate and that the COPSOQ database is built on collected data from surveys performed by the research institute with other organisations, so participants may not be representative of works councils and the working population in general. Furthermore, the meaning and relationship of the person performing the research to the participants is important. So the rate of participation correlates strongly to both, the level of activation and motivation. As the survey was sent out by the administrative centres of trade union IG Metal to work council committees, this could lead to some bias:

At first, it could be assumed that the administrative centers contacted closer related committees. Due to the fact that there is no necessary relationship between both, only committees’ closer to trade unions could have attended. Being focussed on trade union, this group also could attend more often on training courses. As participation was voluntarily, it could be that primary these work councils attended, who suffer from psychosocial stress and strain. Additionally it is to say that, due to the setting of the survey, a partly selected and small sample is compared to relatively extensive groups. Taking these facts into account and adding the missing response rate, there is no claim for representativeness. But focussing the outcomes of this research and comparing the results to the present knowledge about work councils, their specific role in industrial relations and about their burdens [12], it is to say that there are matching results regarding general trends and workload.

## Competing interests

The authors declare that they have no competing interests.

## Authors’ contributions

Design of the study: MR. Recruitment of institutions:MR. Organisation of survey and data collection: MR. Writing of manuscript: MR, MN, SG. All authors have read and approved the final manuscript.

## Pre-publication history

The pre-publication history for this paper can be accessed here:

http://www.biomedcentral.com/1471-2458/12/909/prepub
